# Metabolomics-transcriptomics analysis reveals the mechanism by which *Gentiana lawrencei* var. *farreri* promotes flowering under different diurnal temperatures

**DOI:** 10.3389/fpls.2026.1908845

**Published:** 2026-08-28

**Authors:** Lame Zeren, Zhuoma Deqing, Yue Xu, Haoying Zhang, Yu Zhang, Huiyang Deng, Hongchuan Chen, Rui Gu, Guopeng Chen

**Affiliations:** 1 School of Ethnic Medicine, Chengdu University of Traditional Chinese Medicine, Chengdu, China; 2 School of Pharmacy, Chengdu University of Traditional Chinese Medicine, Chengdu, China

**Keywords:** different diurnal temperatures, flowering, *Gentiana lawrencei* var. *farreri*, metabolomics, transcriptomics

## Abstract

*Gentiana lawrencei* var. *farreri (G. farreri)* is an important endangered Tibetan medicinal plant. This study aimed to uncover temperature-mediated physiological and molecular mechanisms underlying growth and flowering of this endangered Tibetan medicinal herb, providing theoretical guidance for its standardized artificial cultivation. This study established three diurnal temperature treatments: 15/5 °C (S15), 20/10 °C (S20), and 25/15 °C (S25), and conducted integrated analyses of phenotypic, physiological, non-targeted metabolomic, and transcriptomic data. The results showed that S15 significantly promoted plant biomass accumulation and flowering, with a flowering rate of 45.83%, significantly higher than that of other treatments. S15 significantly reduced the levels of soluble sugar, soluble protein, fructose, and starch, while increasing CAT activity to enhance antioxidant capacity; endogenous hormones showed elevated gibberellin (GA) and decreased indole-3-acetic acid (IAA), establishing a hormonal balance favorable for flowering. Integrated metabolomic and transcriptomic analysis revealed that the flavonoid biosynthesis pathway (ko00941) is the core pathway responsive to temperature; S15 induces an increase in the contents of flavonoid metabolites including naringenin, delphinidin, petunidin, and peonidin, and simultaneously upregulates the expression of key genes including the PAL-encoding gene Cluster-62424.2, the 4CL-encoding gene Cluster-45063.0, and the CHS-encoding gene Cluster-44799.3 involved in flavonoid biosynthesis under this condition. S15 reduces the content of osmotic adjustment substances, increases GA content in plant branches, and enhances antioxidant capacity, which synergistically promotes the growth and flowering of *G. farreri*. Additionally, S15 mediates the accumulation of flavonoid compounds by regulating key genes involved in flavonoid biosynthesis, thereby elevating the accumulation of bioactive constituents isoorientin and isoscoparin-2″-O-β-D-glucopyranoside. This study can provide crucial theoretical support for the optimal temperature regulation and control in the artificial cultivation of *G. farreri.*

## 1 Introduction

*Gentiana lawrencei* var. *farreri (G. farreri)* is a perennial herbaceous plant of the family Gentianaceae, genus Gentiana, as one of the mainstream basal species of Tibetan medicine “Bangjian”. Its flowering aerial parts, which lack basal leaves, are used medicinally to treat colds with fever, pneumonia with coughing, and various inflammatory disorders (Yang et al., 2012, Fu Lin et al., 2018, Lin et al., 2024), and is a key raw material for famous Tibetan medicinal preparations such as “Ten Flavors Gentian Flower Granules”, and is also a representative late-flowering plant in the alpine ecosystem (Xiaotong, 2023). This species is primarily distributed in high-altitude regions including Xizang, Sichuan, Qinghai, and Gansu, growing at elevations between 2,410 and 4,600 meters in alpine meadows, shrublands, and floodplains. Its native habitat features ample sunlight, low annual effective accumulated temperature, and a relatively low annual mean temperature (Lin et al., 2024). Currently, the medicinal raw material of *G. farreri* relies entirely on wild resources. Long-term and uncontrolled harvesting has led to a sharp decline in its wild population and an increasing level of endangerment. Therefore, the development of efficient artificial cultivation technology is the way to ensure the sustainable use of the rare Tibetan medicine resources and alleviate the pressure on wild resources. However, artificial cultivation at low elevations exposes plants to excessive ambient temperature, and there has been no systematic study on how temperature changes regulate the growth and development, flowering characteristics, stress resistance, and accumulation of pharmacologically active compounds in *G. farreri*, so the underlying mechanisms remain unclear.

Temperature is a key environmental factor regulating plant flowering. Different plants each have an optimal temperature range for flowering, and abnormal temperatures can significantly suppress the flowering process. Extreme cold not only disrupts plant metabolism and damages plant tissues but can also lead to plant death in severe cases (Xiangrong, 2009).To. adapt to low-temperature stress, plants can alleviate damage by regulating the synthesis and degradation of osmoprotectants, thereby resisting cold-induced injury (Chen et al., 2023). Soluble sugars, soluble proteins, proline, and polyols are key osmoprotectants in plants, and changes in their concentrations are often used as important physiological indicators of plant responses to temperature stress (Chen et al., 2023). Moreover, the dynamic balance of reactive oxygen species in plants is easily disrupted by low temperatures. Plants can activate their enzymatic antioxidant systems to eliminate excess oxidative products and alleviate oxidative damage; this system primarily includes key antioxidant enzymes such as superoxide dismutase (SOD), peroxidase (POD), and catalase (CAT) (Ya, 2008). Previous studies have confirmed that low temperature can significantly alter the endogenous hormone levels in medicinal plants. For example, cold induction increases the accumulation of gibberellin (GA), indole-3-acetic acid (IAA), and abscisic acid (ABA) in Isatis indigotica plants, thereby promoting earlier bolting and flowering (Wu et al., 2022). At present, there is still a lack of systematic research on the specific processes and physiological molecular mechanisms underlying temperature regulation of growth and development in *G. farreri*, as well as the transition from vegetative to reproductive growth.

In addition to physiological responses, temperature can activate complex regulatory networks in plants, triggering systematic changes in gene expression and molecular metabolic processes (Quint et al., 2016). Currently, the molecular mechanisms underlying plant temperature responses have been confirmed in various model plants. Using microarray and transcriptome sequencing technologies, researchers have identified numerous temperature-responsive genes in species such as *Arabidopsis* and rice, and these genes are widely conserved across plants (Sharma et al., 2024). The encoded functional proteins, including ubiquitin ligases, RNA helicases, heat shock proteins, ion transporters, and aquaporins, mediate membrane channel proteins (AQPs, SUTs) in processes such as RNA metabolism, transport of small molecules, and carbohydrate movement, playing a crucial role in plant temperature stress responses (Yoshihiro et al., 2013). Furthermore, plants can maintain cell membrane integrity and prevent acute cellular damage by activating antioxidant signaling pathways and accumulating stress-resistant metabolites including tryptophan, trehalose, flavonoids and glutathione (Mittler, 2002). Meanwhile, stress-related transcription factors such as HSFs, NAC, HSF1, CBF/DREB, and bZIP can respond to temperature signals by binding to specific cis-acting elements like ABRE, DRE/CRT, and HSE, thereby regulating the expression of downstream defense genes (Li et al., 2006, Guo et al., 2011, Li et al., 2021). Most existing studies have focused on model plants and low-altitude crops, while a systematic and comprehensive understanding of the transcriptional-metabolic regulatory mechanisms underlying cold adaptation and flowering in alpine endemic plants remains lacking.

This study aims to investigate the regulatory effects of different diurnal and nocturnal temperatures on the growth, development, and flowering characteristics of *G. farreri*, and to clarify the impact of temperature conditions on the yield and quality formation of its medicinal materials. The experiment involved different diurnal and nocturnal temperature gradients, systematically analyzing plant biomass allocation and flowering phenotypic differences, and measuring soluble sugar, soluble protein, antioxidant enzyme activity, and endogenous hormones. Furthermore, non-targeted metabolomics and transcriptomics were integrated to identify differentially accumulated metabolites (DAMs) and differentially expressed genes (DEGs) under various temperature treatments. By conducting KEGG pathway enrichment analysis and integrated transcriptome-metabolome analysis, we identified core regulatory pathways involved in temperature response, revealing the molecular mechanisms underlying temperature regulation of plant growth, flowering development, and medicinal quality formation in *G. farreri*.

## 2 Materials and methods

### 2.1 Experimental materials and design

The seeds of *G. farreri* used in this experiment were collected in October 2023 from Hongyuan County, Aba Tibetan and Qiang Autonomous Prefecture, Sichuan Province (32°50′42.20″N, 102°35′26.21″E). The experiment was conducted in an artificial climate incubator with a single-factor experimental design, and three diurnal temperature treatments were set up: 15/5 °C (S15), 20/10 °C (S20), and 25/15 °C (S25). The trays (7 cm × 7 cm × 8 cm) were used in the experiment, with approximately 10 seeds sown per tray. Each treatment consisted of 48 trays. After germination, thinning was performed, retaining two uniformly growing seedlings per tray. The experiment was conducted under a 12-hour light/dark cycle, with a light intensity of 40,000 lux and a relative humidity of 65%. Seeds were sown in November 2023, with regular watering supplied throughout the cultivation period during the growing period. Samples were collected 294 days after sowing; non-flowering plants were selected, and nutrient shoots from the same position were harvested to ensure consistent sample tissues.

### 2.2 Morphological indicators and biomass measurement

Ten plants were randomly selected from each treatment to count and record the number of branches, branch length, number of flowers, and flowering time. Additionally, nine uniformly growing plants were selected from each treatment to measure biomass. The entire plant was carefully excavated, rinsed with clean water to remove surface impurities, blotted dry with absorbent paper, and then dried in an 80 °C oven until constant weight was achieved to determine the dry weight of the plant.

### 2.3 Physiological indicators measurement

For each treatment, three randomly selected plant branches were quickly frozen in liquid nitrogen and stored at −80 °C for subsequent physiological measurements. All physiological parameters were determined using corresponding assay kits from Green Biotechnology Co., Ltd., including starch (anthraquinone colorimetric method), sucrose (resorcinol method), soluble sugar (anthraquinone colorimetric method), soluble protein (Coomassie Brilliant Blue G-250 staining method), fructose (resorcinol method), proline (acidic ninhydrin method), malondialdehyde (thiobarbituric acid method), and catalase and peroxidase activities. All measurement procedures were strictly performed according to the instructions provided with the respective kits.

### 2.4 Endogenous hormone content measurement

For each sample, three randomly selected plant branches were processed to determine IAA and GA contents using enzyme-linked immunosorbent assay (ELISA). Accurately weighed 0.05 g of the sample and place it into a pre-cooled mortar. Add 5 mL of isopropanol-HCl extraction buffer and grind thoroughly. Homogenized samples were shaken at 4 °C for 30 minutes, followed by the addition of 10 mL dichloromethane and further shaking at 4 °C for another 30 minutes. The mixture was then centrifuged at 18,000 ×g for 5 minutes at 4 °C, and the lower organic phase was collected. After drying the organic phase under nitrogen gas, it was dissolved in 150 μL methanol containing 0.1% formic acid, filtered through a 0.22 μm membrane for purification, and finally analyzed using a microplate reader to measure absorbance values and calculate IAA and GA concentrations.

### 2.5 Content determination method

The chromatographic analysis was carried out using an Inertsil ODS-3 chromatographic column (4.6 mm × 250 mm, 5 μm), with a mobile phase consisting of 0.2% phosphoric acid aqueous solution (A phase) - methanol (B phase) for gradient elution (0–5 min, 20% → 24% B; 5–42 min, 24% → 38% B; 42–52 min, 38% → 40% B; 52–62 min, 40% → 20% B), at a flow rate of 1.0 mL·min^-^¹, with a detection wavelength of 240 nm, a column temperature of 30 °C, and an injection volume of 10 μL. Under these conditions, all six analytes reached baseline separation (separation degree > 1.5) with the theoretical plate number of gentiopicroside being no less than 6,000. The system suitability was good. Precisely weighed 15.4 mg of loganic acid, 14.98 mg of swertiamarin, 12.81 mg of gentiopicroside, 15.7 mg of sweroside, 16.23 mg of isoorientin, and 14.84 mg of isoscoparin-2″-O-β-D-glucopyranoside, respectively, dissolved in methanol and made up to 10 mL. Then, 1 mL of each reserve solution was precisely transferred to the same 50 mL volumetric flask, made up with methanol, and the mixture of the reference substances was obtained (the mass concentrations of each component were 30.8, 29.96, 25.62, 31.4, 32.46, and 30.76 mg·L^-^¹). The preparation method of the test sample solution was as follows: take about 0.5 g of sample powder (filtered through a 3-mesh sieve), precisely weighed (calculated based on the dry sample, the sample was dried to constant weight in a 60 °C oven before sampling), add 25 mL of methanol, ultrasonic treatment (250 W, 40 kHz) for 30 min, cool down, make up the lost mass with methanol, shake well, and filter to obtain the filtrate. The methodological validation was carried out under the above conditions, and the peak areas of the six components were in good linear relationship with the injection volume within their respective linear ranges (R² ≥ 0.999 6), and the regression equations and linear ranges are shown in **Supplementary Table 2**; in the precision test, the RSD of the peak areas of the six components was less than 2.1% (n = 6), in the stability test, the RSD of the peak areas of the test sample solution within 24 h was less than 2.5% (n = 7), in the repeatability test, the RSD of the content determination was less than 2.8% (n = 6), and in the addition recovery test, the average recovery rates at the low, medium, and high levels were between 97.3% and 102.6%, with RSD less than 2.9% (n = 9), indicating that this method has strong specificity, accuracy, and reliability, and can be used for the determination of the contents of the six triterpenoid glycosides and flavonoid glycosides mentioned above. The sample content was calculated by substituting the peak area into the corresponding regression equation using the external standard method, and the results were expressed in mg·g^-^¹. Under S25 (25 °C/15 °C) treatment, the *G. farreri* plant did not flower or produce flowers during the 294-day experimental period, so there were no flower samples available for HPLC quantitative analysis of the effective components. Under this temperature condition, the plants showed growth stagnation or even death in the later stage of the experiment, indicating that the 25 °C high temperature exceeded its suitable growth range.

### 2.6 Metabolomics analysis

For each sample, three randomly selected plant branches were collected. This study was conducted using an ultra-high performance liquid chromatography-tandem mass spectrometry (UPLC-MS/MS) platform for analysis. The liquid chromatography system was SCIEX ExionLC AD, equipped with a Waters ACQUITY UPLC HSS T3 column (1.8 µm, 2.1 × 100 mm). The mobile phase A was ultrapure water containing 0.1% formic acid, and the mobile phase B was acetonitrile containing 0.1% formic acid. The flow rate was 0.40 mL/min, the column temperature was 40 °C, and the injection volume was 4 µL. The mass spectrometry system was SCIEX TripleTOF 6600+ (Q-TOF), using an electrospray ionization source (ESI), and data was collected in information-dependent acquisition (IDA) mode in both positive ion (ESI+) and negative ion (ESI-) modes. The MS1 mass scan range was 50–1250 Da, and the MS2 mass scan range was 25–1250 Da, with a collision energy of ± 30 V. The quality control (QC) samples were prepared by mixing equal amounts of extracts from each treatment group. One QC sample was inserted between every 10 experimental samples to monitor system stability. The raw mass spectrometry data was converted to mzML format using ProteoWizard, and peak extraction, peak alignment, and retention time correction were performed using the XCMS program. Peaks with a missing rate of more than 50% were filtered, and the remaining missing values were filled using KNN (missing rate < 50%) or 1/5 minimum value (missing rate > 50%). The peak area was corrected using the SVR method. Metabolite identification was completed by searching the self-built database of MivaMetabolomics, integrating public databases, and the met DNA method. Only metabolites with a comprehensive identification score of ≥ 0.5 and a CV value of QC samples < 0.5 were retained for subsequent analysis, resulting in the identification of 2,991 metabolites. The criteria for differentially expressed metabolites were VIP ≥ 1 (based on the OPLS-DA model, verified by 200 random permutation tests) and fold change ≥ 2 or ≤ 0.5. Pathway annotation and enrichment analysis were based on the KEGG database, and the significance of pathways was evaluated using the hypergeometric test.

### 2.7 Transcriptomics analysis

The transcriptome sequencing for this study was completed by Meiyi Metabolism Technology Co., Ltd. Using the Illumina high-throughput sequencing platform for paired-end sequencing (PE150), a total of 9 samples (3 temperature treatments × 3 biological replicates) were analyzed for transcriptome. A cumulative of 79.43 Gb of Clean Data was obtained, with each sample’s Clean Data not less than 7.0 Gb and the Q30 base percentage not less than 96.0%. The original data were filtered by fastp (v0.23.2) for quality control, and then Trinity (v2.15.1) was used for *de novo* assembly without prior knowledge, and Corset (v1.09) was used for hierarchical clustering to remove redundancies. A total of 188,044 Unigene were obtained (average length 1,207 bp, N50 1,712 bp). After filtering the original data with fastp (v0.23.2), the Clean Reads of each sample were aligned to the assembled transcriptome using bowtie2 (v2.4.5), and the gene expression levels (FPKM) and original readcounts were calculated using RSEM (v1.3.1). Differential expression analysis was performed using DESeq2 (v1.22.2), with the threshold of |log_2_Fold Change| ≥ 1 and FDR < 0.05 (Benjamini-Hochberg correction) for screening differentially expressed genes (DEGs). Unigene functional annotation was completed by aligning to KEGG, NR, Swiss-Prot, GO, KOG, TrEMBL, and Pfam databases using DIAMOND BLASTX. Among them, 85,990 genes were annotated by KEGG (accounting for 45.73% of the total Unigene).

### 2.8 qRT-PCR verification

To verify the reliability of the transcriptome data, six key differentially expressed genes in the flavonoid biosynthesis pathway were selected for qRT-PCR validation, including PAL (Cluster-62424.2), C4H (Cluster-60039.3), 4CL (Cluster-45063.0), CHS (Cluster-44799.3), F3’5’H (Cluster-64204.0), and DFR (Cluster-44367.0), covering the entire pathway from the upstream phenylpropanoid pathway entry to the downstream anthocyanin branch. Using total RNA from samples corresponding to the transcriptome sequencing as the template, cDNA was synthesized using the PrimeScript RT reagent Kit (TaKaRa). The qRT-PCR reaction was performed on the real-time fluorescence quantitative PCR instrument, using the SYBR Green Premix Ex Taq II (TaKaRa) reagent kit. The reaction system was 10 µL, with 3 biological replicates and 3 technical replicates for each group. Actin was used as the internal reference gene, and the relative expression level was calculated using the 2^(−ΔΔCt) method, with S15 as the calibration sample (Calibrator). The primer sequences are shown in Supplementary Material **Supplementary Table 1**.

### 2.9 Statistical analysis

All data were organized and summarized using Excel 2019 software, and graphing was performed using GraphPad Prism 10 software. Statistical significance was analyzed by one-way ANOVA followed by Duncan’s multiple range test (*P* < 0.05).

## 3 Results

### 3.1 Biomass of each organ

**Figure 1:**
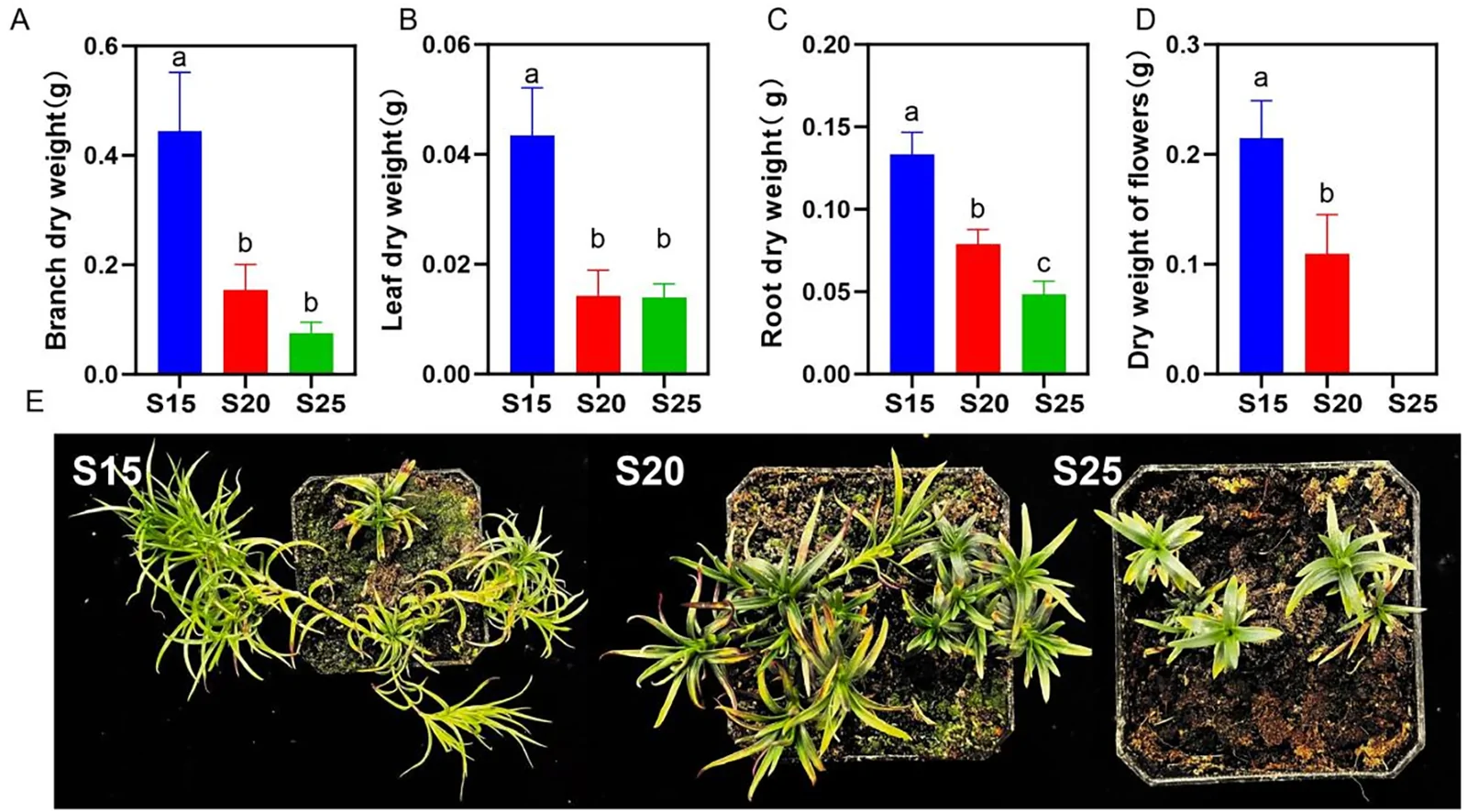
Effects of different diurnal temperature regimes on the biomass of *G. farreri*. **(A)** Branch dry weight, **(B)** leaf dry weight, **(C)** root dry weight, **(D)** flower dry weight, **(E)** growth status. S15, S20 and S25 represent plants grown under different diurnal temperature conditions, corresponding to 15/5 °C, 20/10 °C and 25/15 °C, respectively. Different lowercase letters indicate significant differences between groups (*P* < 0.05).

S15 significantly promoted the growth of *G. farreri* (**Figure 1E**). Plant morphology varied markedly under different diurnal temperature conditions, particularly in terms of stem elongation, internode length, and leaf shape. Under S15 treatment, stems were the longest, with slightly elongated basal internodes and tightly clustered apical internodes, and leaves ranged from linear to narrowly lanceolate. Stems under S20 treatment were slightly shorter, while other morphological characteristics were largely similar to those under S15. Under S25 treatment, stems were significantly shortened, and some plants failed to produce branches. Dry weights of branches, leaves, roots, and flowers were all significantly higher under S15 compared to S20 and S25 (**Figures 1A–D**), indicating that S15 favored dry matter accumulation and allocation in *G. farreri*.

### 3.2 Flowering characteristics and endogenous hormone contents

**Figure 2:**
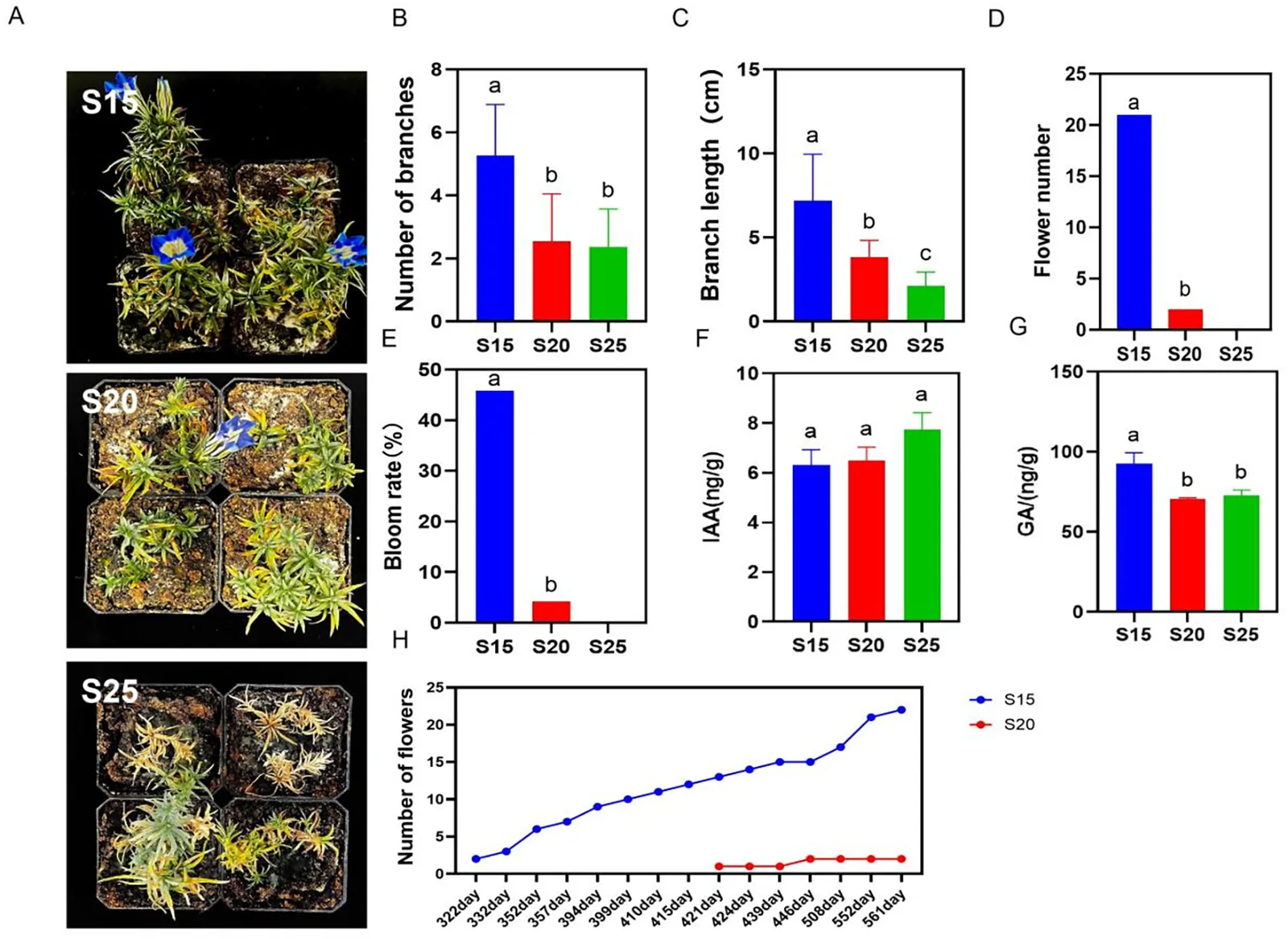
Effects of different diurnal temperatures on flowering traits and endogenous hormone contents of *G. farreri*. **(A)** Flowering phenotype of *G. farreri*, **(B)** number of branches, **(C)** branch length (cm), **(D)** number of flowers, **(E)** flowering rate (%), **(F)** IAA content, **(G)** GA content, **(H)** flowering time. S15, S20 and S25 represent plants grown under different diurnal temperature regimes of 15/5 °C, 20/10 °C and 25/15 °C, respectively. Different lowercase letters indicate significant differences between groups (*P* < 0.05).

The branch number, branch length and flower number of S15 were all significantly higher than those of S20 and S25 (**Figures 2B–D**). Compared with S20, S15 exhibited a 94.11% increase in branch number, an 89.15% increase in branch length, and a 600% increase in flower number; compared with S25, the branch number of S15 increased by 120% and the branch length increased by 290%. Under S15 treatment, *G. farreri* flowered 322 days after sowing, while plants treated with S20 flowered 421 days after sowing, and no flowering was observed in the S25 treatment (**Figure 2H**). The Flowering rate of S15 reached 45.83%, while that of S20 was only 4.17% (**Figure 2E**). GA content in S15 was significantly higher than that in S20 and S25, and IAA content was significantly lower than that in S25 (**Figures 2F, G**).

### 3.3 Contents of osmoprotective substances and activities of antioxidant enzymes

**Figure 3:**
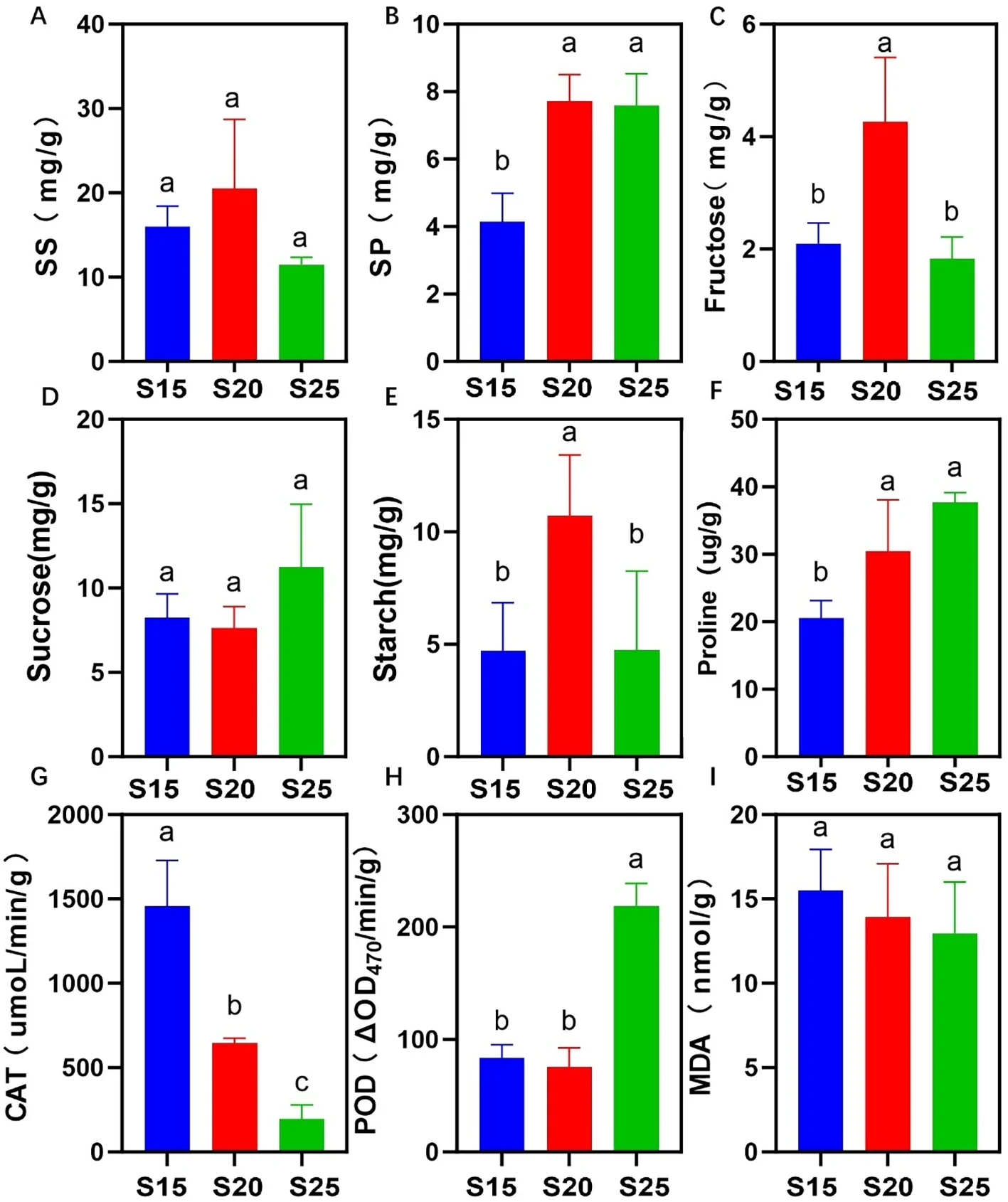
Effects of different diurnal temperatures on physiological indicators of *G. farreri*. **(A)** Soluble sugar content, **(B)** soluble protein content, **(C)** fructose content, **(D)** sucrose content, **(E)** starch content, **(F)** proline content, **(G)** CAT enzyme activity, **(H)** POD enzyme activity, **(I)** MDA mass fraction. SS: soluble sugar, SP: soluble protein. S15, S20, and S25 represent plants grown under different day-night temperature conditions, corresponding to 15/5 °C, 20/10 °C, and 25/15 °C, respectively. Different lowercase letters indicate significant differences between groups (*P* < 0.05).

The contents of soluble sugar, soluble protein, fructose and starch in S15 were all significantly lower than those in S20, and the contents of soluble protein and starch were significantly lower than those in S25 (**Figures 3A–E**). There was no significant difference in MDA content among the various temperature treatment groups (**Figure 3I**), indicating that the S15 treatment did not cause obvious membrane lipid peroxidation damage. The CAT activity of S15 was significantly higher than that of S20 and S25, while the POD activity was significantly lower than that of S25 (**Figures 3G, H**). Under S15 conditions, plants can efficiently scavenge reactive oxygen species (ROS) by increasing CAT activity, maintain *in vivo* redox homeostasis, and provide a stable physiological environment for the flower bud differentiation of *G. farreri*.

### 3.4 Content of active ingredients under different diurnal temperatures

**Figure 4:**
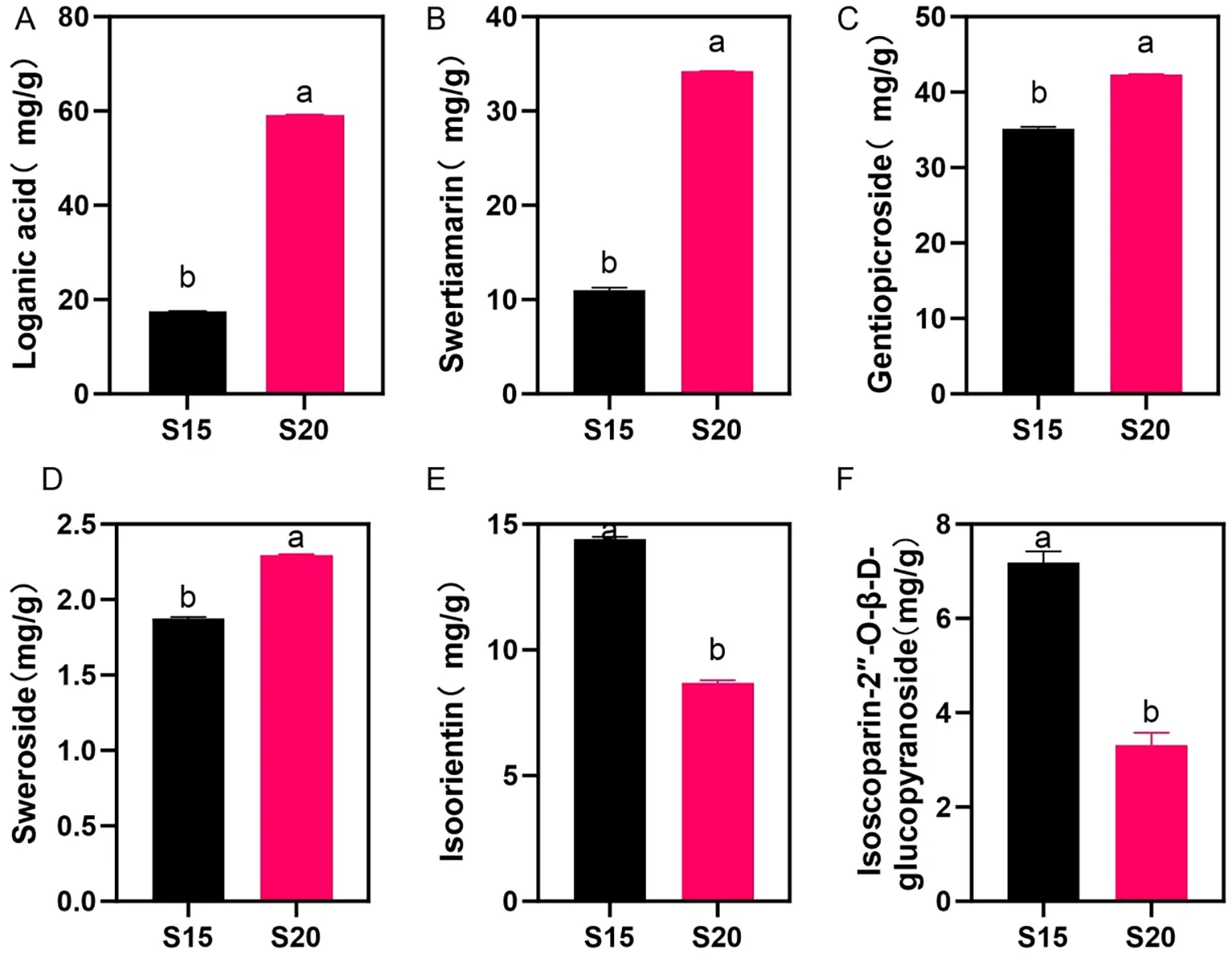
Content of active components under different diurnal temperatures. **(A)** Loganic acid, **(B)** swertiamarin, **(C)** gentiopicroside, **(D)** sweroside, **(E)** isoorientin, **(F)** isoscoparin-2″-O-β-D-glucopyranoside. S15 and S20 represent plants grown under different day-night temperature conditions, which are 15/5 °C and 20/10 °C, respectively. Different lowercase letters indicate significant differences between groups (*P* < 0.05).

The Loganic acid (17.55 mg/g), Swertiamarin (10.81 mg/g), Gentiopicroside (35.18 mg/g), and Sweroside (1.88 mg/g) in S15 were significantly lower than those in S20. Meanwhile, Isoorientin (14.46 mg/g) and Isoscoparin-2″-O-β-D-glucopyranoside (7.07 mg/g) were significantly higher than those in S20 (**Figures 4A–F**).

### 3.5 Metabolomic analysis of *G. farreri* in response to different diurnal temperatures

**Figure 5:**
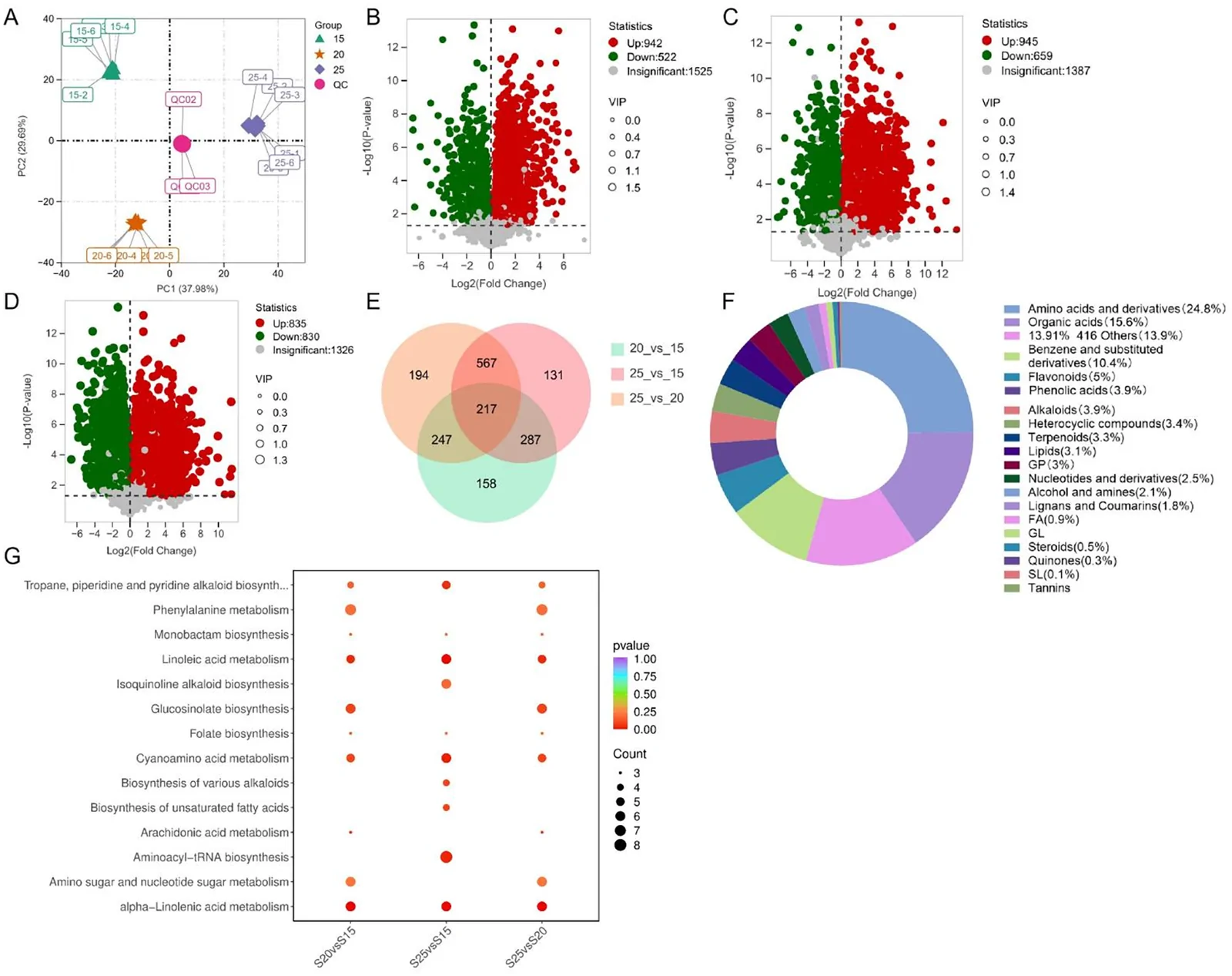
Metabolomics analysis of *G. farreri* under different day-night temperature treatments. **(A)** PCA analysis of all samples, **(B)** Volcano plot of differential metabolites between S20 and S15, **(C)** Volcano plot of differential metabolites between S25 and S15, **(D)** Volcano plot of differential metabolites between S25 and S20, **(E)** Venn diagram, **(F)** Statistical analysis of the proportional distribution of metabolome chemical structure classification, **(G)** KEGG enrichment pathway. S15, S20 and S25 represent plants grown under different diurnal temperature conditions, which are 15/5 °C, 20/10 °C and 25/15 °C, respectively.

To evaluate the differential metabolites of *G. farreri*, multivariate statistical analysis was performed. The results of principal component analysis (PCA) showed that there were significant differences among the S15, S20 and S25 samples, with PC1 and PC2 accounting for 37.98% and 29.69% of the total variation, respectively (**Figure 5A**). In addition, samples clustered tightly within each group, showing good experimental repeatability. To detect changes in metabolite expression patterns across different treatments, we analyzed the metabolic differences among S15, S20, and S25, and identified DAMs based on the criteria of VIP ≥ 1.0 and P ≤ 0.05. A total of 2991 metabolites were identified. Between S20 and S15, 1464 significant DAMs were identified, among which 942 DAMs were significantly higher abundance and 522 DAMs were significantly lower abundance (**Figure 5B**). Between S25 and S15, 1604 significant DAMs were identified, among which 945 DAMs were significantly higher abundance and 659 DAMs were significantly lower abundance (**Figure 5C**). Between S25 and S20, 1665 significant DAMs were identified, among which 835 DAMs were significantly higher abundance and 830 DAMs were significantly lower abundance (**Figure 5D**), and 217 overlapping DAMs were detected (**Figure 5E**).

Based on their structural characteristics, these metabolites were classified into 20 distinct chemical classes (**Figure 5F**). Among them, the dominant classes include amino acids and their derivatives (accounting for 24.8%), organic acids (15.6%), benzene and its derivatives (10.4%), flavonoids (5%), phenolic acids (3.9%), alkaloids (3.9%), heterocyclic compounds (3.4%), terpenoids (3.3%) and lipids (3.1%). To further investigate the effects of different treatments on the metabolic pathways of *G. farreri*, KEGG pathway enrichment analysis was performed on the identified DAMs (**Figure 5G**). These DAMs were significantly enriched in three pathways: linoleic acid metabolism (ko00591), α-linolenic acid metabolism (ko00592), and flavonoid biosynthesis (ko00941).

### 3.6 Transcriptomic analysis of *G. farreri* in response to different diurnal temperatures

**Figure 6:**
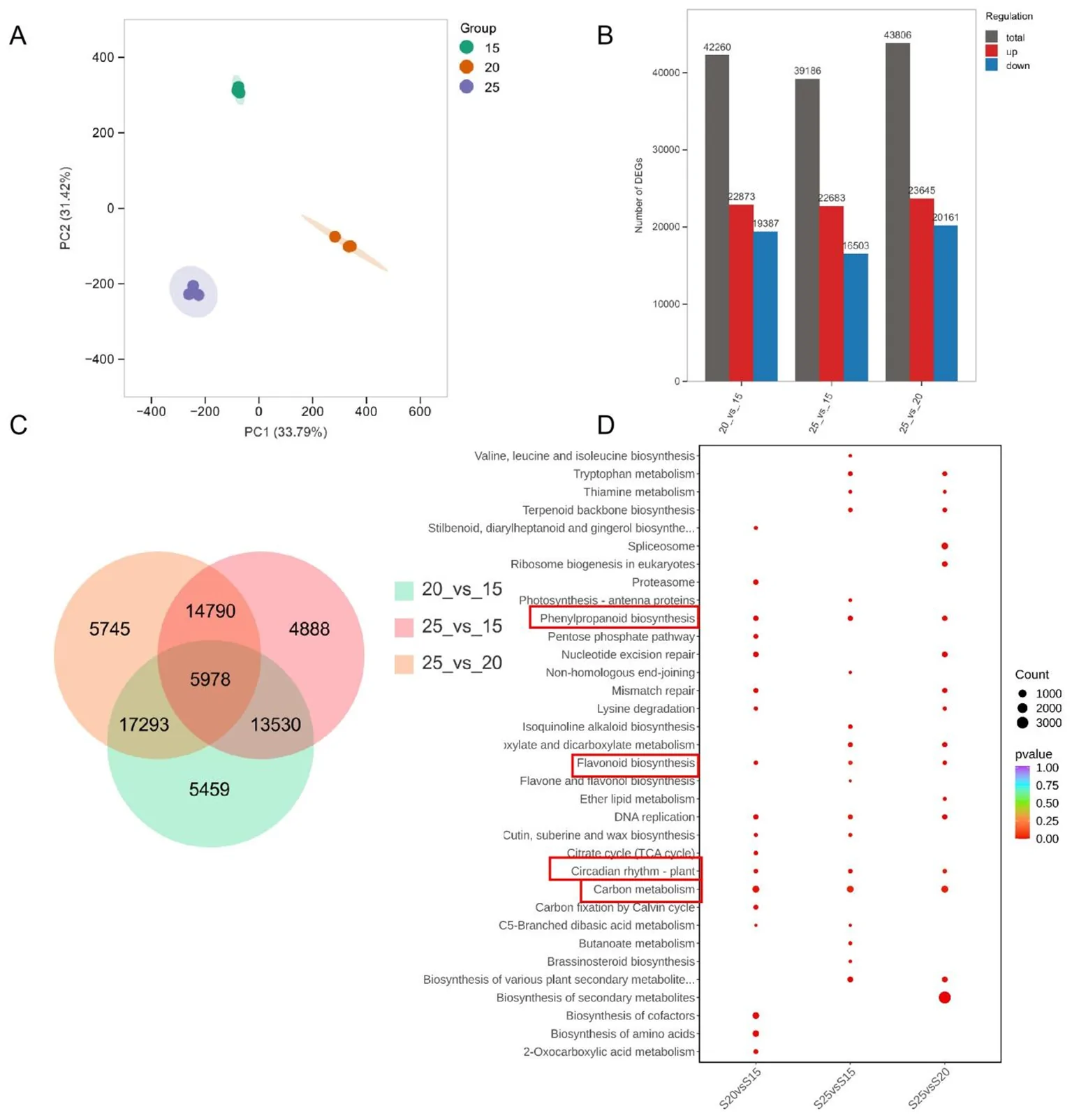
Transcriptome analysis of *G. farreri* in response to different diurnal temperatures. **(A)** PCA, **(B)** histogram of the number of differentially expressed genes, **(C)** Venn diagram, **(D)** KEGG enriched pathways. S15, S20 and S25 represent plants grown under different diurnal temperature conditions, which are 15/5 °C, 20/10 °C and 25/15 °C, respectively.

DEGs in the test samples were identified to investigate the molecular response of *G. farreri* to varying diurnal temperatures. PCA was performed on the transcriptome data of 9 samples, and the results revealed significant differences among samples S15, S20 and S25, with PC1 and PC2 explaining 33.79% and 31.42% of the total variation, respectively (**Figure 6A**). In addition, the tight clustering of samples within each group indicates good experimental reproducibility and high data quality.

Under different diurnal temperature treatments, *G. farreri* exhibits significant differences in gene expression. Accordingly, we examined gene expression in these populations under varying day-night temperature conditions. A strict threshold of |log2Fold Change| ≥ 1 and FDR < 0.05 was applied to assess the significance of differential gene expression. The results show that 42260 DEGs, including 22873 higher abundance genes and 19387 lower abundance genes, were identified in the comparison group S20 vs S15; 39186 DEGs (22683 higher abundance, 16503 lower abundance) were detected in S25 vs S15; and 43806 DEGs (23645 higher abundance, 20161 lower abundance) were obtained in S25 vs S20. In all comparisons, the number of higher abundance genes exceeds that of lower abundance genes (**Figure 6B**). The distribution of DEGs across these comparison groups is visualized via a Venn diagram (**Figure 6C**): 13530 common DEGs were identified between S20 vs S15 and S25 vs S15, 17293 common DEGs between S20 vs S15 and S25 vs S20, and 14790 common DEGs between S25 vs S20 and S25 vs S15.

To further characterize the functions of DEGs, KEGG pathway enrichment analysis was performed. The results (**Figure 6D**) showed that Flavonoid biosynthesis (ko00941), Circadian rhythm - plant (ko04712), and Carbon metabolism (ko01200) were all significantly differentially enriched, indicating that there exist regulatory differences at the transcriptional level in metabolic processes among different treatment groups. Genes associated with Flavonoid biosynthesis were significantly lower abundance in the comparison of S25 vs S20; genes related to Carbon metabolism were significantly lower abundance in the comparison of S20 vs S15. These results suggest that genes related to carbon metabolism and flavonoid biosynthesis synergistically respond to temperature changes at the transcriptional level.

### 3.7 Integrated analysis of transcriptome and metabolome

**Figure 7:**
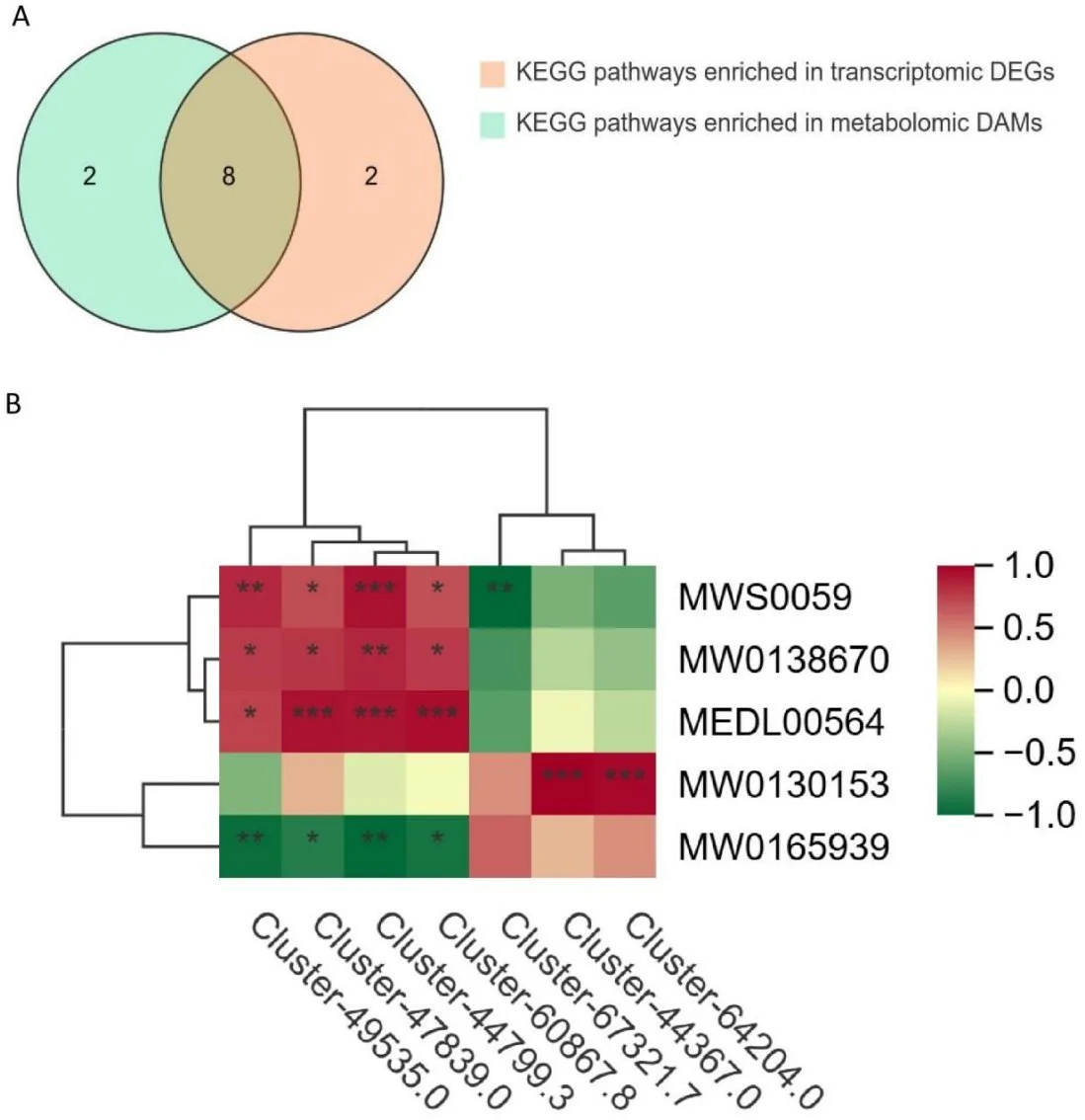
Integrated analysis of transcriptome and metabolome of *G. farreri*. **(A)** Venn diagram of the intersection of KEGG enrichment pathways for differentially expressed genes (DEGs) and differentially accumulated metabolites (DAMs); **(B)** Spearman correlation heatmap of key genes in the flavonoid biosynthesis pathway (ko00941) and differential flavonoid metabolites. MW0130153 (Taxifolin 3-O-bata-xylopyranoside), MW0138670 (kaempferol 7-O-glucoside), MW0137389 (Apigenin triacetate), MEDL00564 (Cynaroside), MW0127606 (Epigallocatechin 3,3’,-di-O-gallate), MW0165939 (cyanidin 3-O-glucoside-7-O-(6-O-(p-hydroxybenzoyl)-glucoside)). The color of the heatmap ranges from green to red, indicating that the correlation changes from weak to strong, and asterisks denote significant correlation: *** *P* < 0.001, ** *P* < 0.01, * *P* < 0.05.

The results of combined transcriptomic and metabolomic analysis showed that there was a significant overlap in the KEGG enrichment pathways between DEGs and DAMs. The Venn diagram analysis (**Figure 7A**) indicated that the two omics approaches jointly identified 8 shared enriched pathways, among which the flavonoid biosynthesis pathway (ko00941) was the core pathway. This pathway is significantly affected by temperature treatment at both transcriptional and metabolic levels. The molecular differences before flower bud differentiation are associated with the timing of flowering, making it a key target for understanding the temperature response and flowering mechanism of *G. farreri.*

Further correlation heatmap analysis revealed (**Figure 7B**) that the key structural genes in the flavonoid pathway (including FLS, LDOX, CHS, ANS, DFR, and F3’5’H) were all extremely significantly correlated with differential flavonoid metabolites (including rutin, kaempferol, luteolinic acid, quercetin, and anthocyanidin) (P < 0.001). Under the S15 condition, the expression of key genes in this pathway was significantly positively correlated with the accumulation of flavonoid metabolites.

### 3.8 Genes and metabolites involved in flavonoid biosynthesis

**Figure 8:**
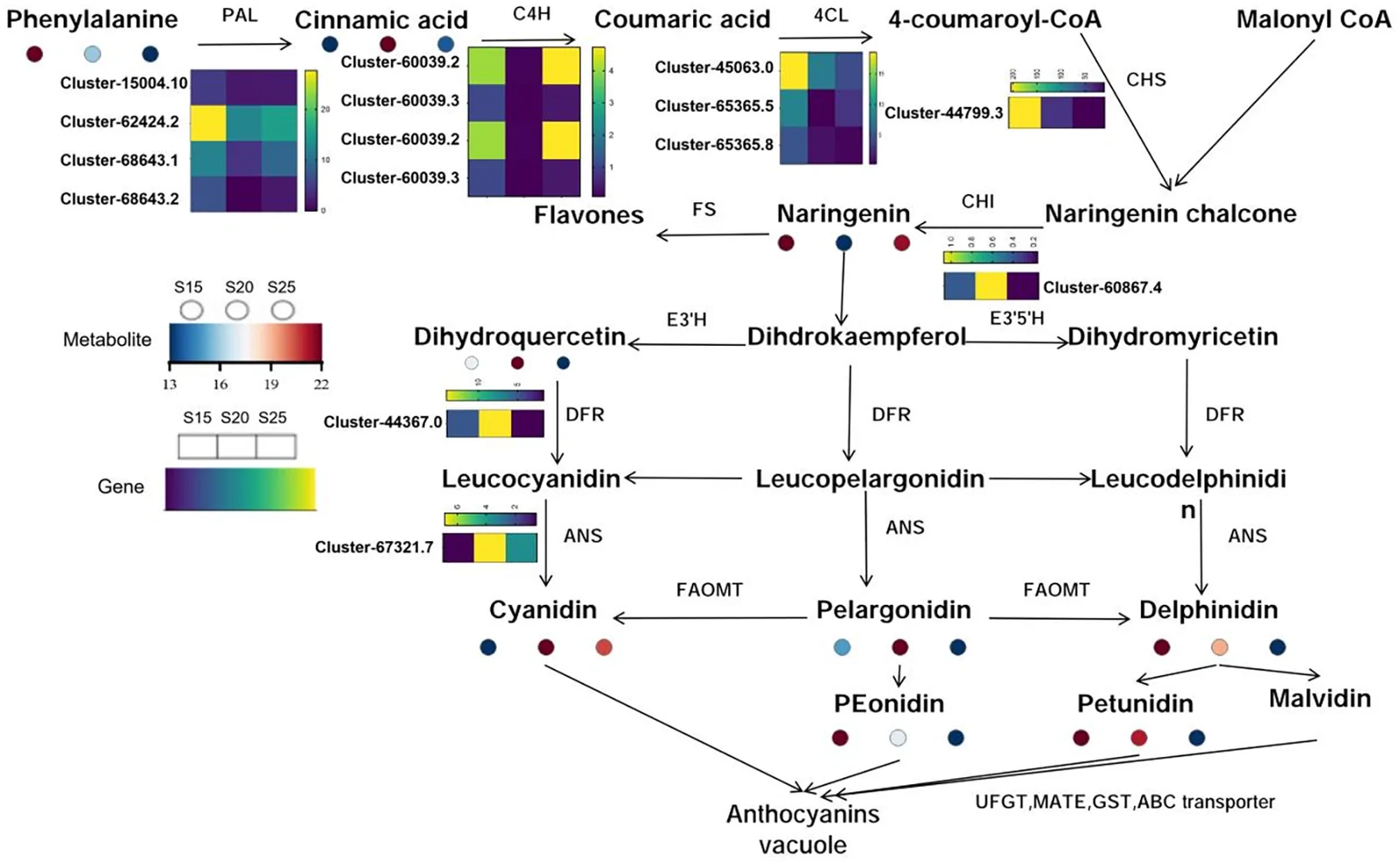
Heatmap of the expression of key genes and metabolites involved in the flavonoid biosynthesis pathway. Circles represent metabolite accumulation levels, and squares represent gene expression levels. PAL: Phenylalanine ammonia-lyase, C4H: Cinnamate 4-hydroxylase, 4CL: 4-Coumarate: CoA ligase, CHS: Chalcone synthase, CHI: Chalcone isomerase, F3’H: Flavonoid 3’-hydroxylase, F3’5’H: Flavonoid 3’,5’-hydroxylase, DFR: Dihydroflavonol 4-reductase, ANS: Anthocyanidin synthase. S15, S20 and S25 denote plants grown under different diurnal temperature conditions, corresponding to 15/5 °C, 20/10 °C and 25/15 °C, respectively.

Temperature treatment significantly regulates flavonoid biosynthesis in the branches of *G. farreri.* To intuitively illustrate the characteristics of gene expression and metabolite changes in the flavonoid biosynthesis pathway under temperature gradient treatments, a metabolic pathway map was constructed (**Figure 8**). The results showed that the contents of naringenin, delphinidin, petunidin and peonidin all increased gradually as temperature decreased; the expression levels of key genes including the PAL-encoding gene Cluster-62424.2, the 4CL-encoding gene Cluster-45063.0, and the CHS-encoding gene Cluster-44799.3 were also synchronously higher abundance with decreasing temperature. The S15 can activate the flavonoid biosynthetic pathway and promote metabolite accumulation by increasing the expression of key genes.

### 3.9 qRT-PCR verification

To verify the reliability of the transcriptome data, qRT-PCR analysis was conducted on six key genes of the flavonoid biosynthesis pathway (PAL, C4H, 4CL, CHS, F3’5’H, DFR). The results showed that the qRT-PCR expression trends were highly consistent with the transcriptome FPKM data: PAL, C4H, 4CL, and CHS had the highest expression levels under the S15 low-temperature condition, and gradually decreased as the temperature increased; F3’5’H and DFR reached their peak under the S20 moderate-temperature condition. Pearson correlation analysis indicated a significant positive correlation between the log_2_ fold change of FPKM and qPCR (R = 0.87, P < 0.01), confirming the reliability of the transcriptome data (**Supplementary Figure 1**).

## 4 Discussion

### 4.1 Temperature is a critical environmental factor that promotes flowering in alpine medicinal plants

Temperature is a key environmental factor that regulates plant growth and development as well as the transition from vegetative growth to reproductive growth. It not only determines the geographic distribution pattern of alpine plants, but also regulates plant metabolism and developmental processes via signaling pathways, which is of critical significance for the environmental adaptation and population reproduction of endemic plant species on the Qinghai-Tibet Plateau (Wagner et al., 2011; Feng et al., 2025). The alpine habitat is characterized by perennial low temperature, large diurnal temperature difference and short growing season, and such unique environmental conditions have driven the evolution of specific temperature response mechanisms in native plants. *G. farreri*, a typical alpine herbaceous species native to the plateau, has its growth and flowering highly dependent on low-temperature signals, which is a core evolutionary strategy formed through long-term adaptation to the harsh high-altitude environment (Hou et al., 2009, Shi et al., 2023, Feng et al., 2025).

Appropriate temperature can regulate photosynthetic carbon fixation, substance allocation and energy metabolism in plants, and determine the growth rate of plants and the initiation process of flowering. Temperature conditions consistent with the plant’s native habitat can reduce plant energy consumption, improve the utilization rate of photosynthetic products, and promote biomass accumulation and reproductive organ development (Tao et al., 2012). This study reveals that the temperature regime of 15/5 °C (S15) can activate the flowering pathway of *G. farreri*, optimize the carbon allocation pattern, significantly increase the number of branches, the number of flowers and the flowering rate of plants, and advance flowering time (**Figures 2B–H**), confirming that temperature is a necessary condition for *G. farreri* to initiate the flowering program. Although the flowering of numerous alpine plant species requires low temperatures, under moderately warm conditions (25/15 °C), the extent to which flowering is completely inhibited varies among species. For instance, some high-altitude Rhododendron species can still flower partially under warmer conditions (Winarni et al., 2016), whereas flowering of *G. farreri* is completely inhibited, indicating that this species has an especially strict dependence on low temperatures. These results are consistent with the research conclusions that most alpine plants depend on low temperature to complete reproductive conversion (Zhang et al., 2022; Wunder et al., 2023), indicating that floral bud differentiation of *G. farreri* exhibits an obligate low-temperature requirement, which is also an important adaptive trait distinguishing it from low-altitude plants. In contrast, S25 treatment disrupts the photosynthetic metabolism of plants, hinders the transport of nutrients to reproductive organs, and completely inhibits plant flowering, which conversely confirms the necessity of proper growth temperature for its flowering.

Under the treatment S15, the biomass of all organs of the plant increased significantly. Existing studies have shown that S15 can reduce the respiratory consumption of plants, improve net photosynthetic efficiency, regulate the allocation of photosynthetic products, reduce the translocation of photosynthetic products to roots, and promote their accumulation in shoots. While ensuring robust vegetative growth, this process provides sufficient material basis for flowering and reproduction (Wang et al., 2024). Results of hormone regulation analysis showed that under the S15 condition, the level of gibberellin (GA) increased and the level of indole-3-acetic acid (IAA) decreased (**Figures 2F, G**), and this hormonal change is conducive to flowering (Huang, 1996; Zhang et al., 2018). Specifically, GA promotes the floral transition of apical meristems, while the decrease in IAA content relieves its inhibitory effect on flower bud differentiation. The two factors synergistically initiate the flowering process of plants (Zhong et al., 2020). In conclusion, the diurnal temperature conditions adapted to the plateau habitat are the core environmental factor that induces flower bud differentiation of *G. farreri* and ensures its normal flowering and reproduction.

### 4.2 Effects of temperature on osmotic regulatory substances and antioxidant enzyme activities of alpine plants

Plants respond to temperature changes mainly through two major pathways, namely remodeling the homeostasis of osmotic regulators and activating the antioxidant system, which synergistically maintain cell membrane integrity, ROS homeostasis and metabolic stability (Mittler, 2002; Wahid et al., 2007). Among these, osmotic regulators can regulate cellular osmotic pressure and participate in temperature signal transduction, while antioxidant enzymes such as CAT and POD can scavenge excess ROS and alleviate oxidative damage. Together, these two pathways jointly determine the temperature adaptability and growth homeostasis of plants (Bita and Gerats, 2013).

The results of this study demonstrate that under the condition S15, the contents of osmotic adjustment substances including soluble sugar, soluble protein and starch in *G. farreri* are significantly lower than those under S25 (**Figures 3A–E**). Under the S15 condition, no severe irreversible stress signs were observed. However, the decrease in osmotic adjustment substances should be regarded as the result of a change in the priority of energy allocation in the plant, rather than direct evidence of “no stress”. Meanwhile, The increase in CAT activity observed after S15 treatment suggests a potential response of the antioxidant system. However, this result is limited and does not provide sufficient evidence to determine whether the ROS homeostasis has been effectively maintained. Further comprehensive evaluation involving multiple indicators is required in the future. This physiological pattern characterized by “low osmotic substance accumulation and high CAT antioxidant activity” is the core strategy for *G. farreri* to adapt to low-temperature habitats in plateaus, and achieves energy balance between stress resistance defense and growth and reproduction. In addition, the differences in plant osmotic metabolism and oxidative homeostasis shaped by different temperatures also provide an important physiological basis for the subsequent differential expression of genes and metabolites.

### 4.3 Integrated transcriptomic and metabolomic responses and flowering regulation mechanism of *G. farreri* at S15

In this study, we systematically characterized the molecular response of *G. farreri* to S15 via integrated transcriptomic and metabolomic analyses. We identified that the flavonoid biosynthesis pathway acts as the core pathway mediating S15 regulation of plant growth and flowering, and confirmed that S15 can regulate the flowering and developmental process of *G. farreri* by synergistically activating the flavonoid synthesis pathway. Metabolomic results showed that core flavonoid metabolites including3quercetin, kaempferol, rutin and anthocyanins were significantly3enriched under S15 conditions. Meanwhile, the contents of the active components isoorientin and isoscoparin-2″-O-β-D-glucopyranoside also continuously accumulated as temperature decreased, indicating that S15 positively regulates flavonoid metabolism. KEGG enrichment analysis demonstrated that the flavonoid biosynthesis pathway (ko00941) is the core secondary metabolic pathway for temperature response; meanwhile, linoleic acid metabolism and α-linolenic acid metabolism were also significantly enriched, which provides important physiological guarantees for low-temperature adaptation and floral transition of *G. farreri* by maintaining the structural stability of cell membranes (Wang and L., 2014).

Transcriptome analysis further confirmed that flavonoid biosynthesis, plant circadian rhythm, and carbon metabolism are the core pathways under different temperature treatments. S15 can significantly induce high expression of key flavonoid biosynthesis genes including PAL, 4CL, and CHS, while high temperature environment significantly inhibits the transcription level of genes in the aforementioned pathways. This redirects the metabolic resources of the plant to stress defense, disrupts the normal rhythm of vegetative growth and reproductive development, and ultimately leads to the inhibition of flowering. Integrative transcriptome and metabolome association analysis revealed that the key genes of the flavonoid pathway are significantly and positively correlated with the corresponding differential metabolites under the S15 condition, forming a stable gene-metabolite co-regulatory module. This further verifies that the flavonoid pathway acts as a core molecular hub linking low-temperature environmental signals to the floral development of *G. farreri* (Zhao et al., 2021, Zhou et al., 2021).

Previous studies have confirmed that CBF/DREB1 transcription factors can bind to the cis-acting elements in the promoter region of the CHS gene and regulate the transcriptional expression of downstream genes. The up-regulated key genes including CHS identified in this study may be regulated by signaling in this pathway (Tang et al., 2020; Gao et al., 2025). As the rate-limiting gene for flavonoid synthesis, CHS directly determines the metabolic flux of the pathway, and can be used as a key molecular marker for evaluating the flowering potential of *G. farreri* (Hussain et al., 2023, Yang et al., 2025).

In this study, the components of iridoid glycosides (e.g., chemonine, sanguinarine, gentiopicroside, and sanguinarine glycoside) and flavonoid glycosides (e.g., isohymenopterone, isomelilotin-2”-O-pyran glucose) exhibited opposite accumulation trends under the S15 condition - the former significantly decreased while the latter continued to accumulate. This differentiated metabolic response pattern suggests that S15 regulates the secondary metabolites of the lineleaf gentian through pathway specificity: flavonoid components, as the terminal products of the phenylpropanoid metabolic pathway, their accumulation is enhanced through the coordinated upregulation of upstream key genes such as PAL, 4CL, and CHS induced by low temperature; while iridoid glycosides, as the downstream branch products of the terpenoid indole alkaloid synthesis pathway, their content decreased under low temperature possibly due to the prioritization of carbon precursors flowing to the flavonoid pathway rather than the terpenoid pathway under resource-limited conditions, and it may also involve the accelerated conversion or degradation of iridoid glycosides under low-temperature conditions. This phenomenon has been reported in some studies on low-temperature stress in medicinal plants (Miettinen et al., 2014; He et al., 2024; Yin et al., 2024), indicating that different types of secondary metabolites may participate in the plant’s low-temperature adaptation and flowering initiation process through differentiated regulatory strategies in response to low-temperature signals.

Based on literature speculation, In the downstream flowering regulatory network, flavonoids synergistically regulate the flowering process mainly through three core pathways: First, by inhibiting the expression of the flowering repressor gene FLC, the transcriptional silencing of core flowering genes such as FT, SOC1 and LFY is relieved, and the floral bud differentiation program is initiated (Helliwell et al., 2006, Ormancey and Qüesta, 2025); Second, it optimizes the endogenous GA/IAA hormone balance in plants, amplifies the low-temperature flowering signal, and promotes the transition to reproductive growth (Rusak and Černi, 2006, Shi et al., 2023); Third, it maintains intracellular ROS homeostasis, ensures the normal transport of FT flowering protein to the apical meristem, stabilizes the output of flowering signals, and guarantees the smooth progression of the flowering program (Corbesier et al., 2007; Huang et al., 2025).

In conclusion, this study systematically elucidates the proper temperature flowering regulatory mechanism of *G. farreri* by integrating plant phenotypic characteristics, physiological response profiles, and transcriptomic and metabolomic data. A regulatory model is proposed, which specifies that proper temperature signals induce the activation of the flavonoid pathway, collaboratively optimize endogenous hormone homeostasis and ROS steady-state, and ultimately promote the flowering and development of plants. This study reveals the core evolutionary mechanism underlying the adaptation of *G. farreri* to high-altitude habitats and the completion of natural reproduction, and also provides important theoretical basis and key candidate gene resources for the artificial cultivation, precise flowering regulation, and conservation and utilization of wild resources of characteristic alpine medicinal plants.

## 5 Conclusions

This study systematically analyzed the effects of different diurnal temperature regimes on the growth, flowering, physiological metabolism and molecular regulation of *G. farreri* Franch. S15 (15/5 °C) was found to facilitate the growth and flowering of *G. farreri*, and significantly increased biomass, flowering rate, branch number and flower number. S15 promoted floral bud differentiation via hormone balance characterized by elevated gibberellic acid (GA) and reduced indole-3-acetic acid (IAA), maintained oxidative homeostasis by increasing catalase (CAT) activity, and optimized energy allocation. The flavonoid biosynthesis pathway (ko00941) was identified as the core pathway underlying S15 promoted flowering. S15 upregulates the expression levels of key genes encoding PAL (Cluster-62424.2), 4CL (Cluster-45063.0), and CHS (Cluster-44799.3), and synergistically promotes the accumulation of flavonoid metabolites such as naringenin, Delphinidin, Petunidin, and Peonidin. The content of active ingredient Isoorientin and Isoscoparin-2″-O-β-D-glucopyranoside increases, while the contents of active ingredients such as mangelin acid, sanguinarine, gentiopicroside, and sanguinarine decrease. Collectively, S15 promotes flowering in *G. farreri* through the synergistic activation of hormone regulation, antioxidant adaptation and the flavonoid biosynthesis pathway. Despite these findings, this study still has limitations: it lacks direct genetic evidence, such as data from mutants or transgenic lines. Future studies should adopt gene silencing or overexpression technologies in Gentiana plants to verify this causal relationship.

## Data Availability

The datasets generated and analyzed during the current study are available in the National Center for Biotechnology Information (NCBI) repository under the accession number PRJNA1438961. Query link: http://www.ncbi.nlm.nih.gov/bioproject/1438961.
