# Supplementary material for: Metabolomics-transcriptomics analysis reveals the mechanism by which Gentiana lawrencei var. farreri promotes flowering under different diurnal temperatures
Source: Front Plant Sci. 2026 Aug 28;17:1908845. doi: 10.3389/fpls.2026.1908845 (PMC13561937; doi:10.3389/fpls.2026.1908845)
Supplement: Supplementary file 1 [file Table1.docx]

**Supplementary Material**

**Table S1** Primers for qRT-PCR for different genes

|  | Forward 5’-3’ | Reverse 5’-3’ |
| --- | --- | --- |
| Primers used in vector construction and sequencing | | |
| *Cluster-62424.2* | CGGAGTGAAGGCTAGTAGCG | CGACCCAACTGCTGTACCAT |
| *Cluster-45063.0* | GGCTAAGGTGCCGTTTTGTG | CGTCACCTAGGCGGTTTCTT |
| *Cluster-44799.3* | TTCGGTGAAGCGGTTCATGA | GGCTTCTAGGCCGAGTGTTT |
| *Cluster-64204.0* | TTCACATTGAGCCGAGTCCC | TGAACGCCAACAAAAGCCAG |
| *Cluster-44367.0* | AATTAGGCTGGGTGGGAACG | TGTTCGTGATCCCGGGAATC |
| *Cluster-60039.3* | GGACATGAGAGGAGAGCTGC | GTGTTTACCACTCCCCTGCA |

**Fig. S1** Quantitative real-time PCR (qRT-PCR) validation of key genes in the three groups. Blue squares: the FPKM values obtained from transcriptome analysis; Red circles: the relative expression levels determined by qRT-PCR. Data are shown as mean ± SE (n = 3).

**Table S2** Linear Regression Equations and Linear Ranges of the Active Components

| Compound | regression formula | R^2^ | linear range/ug |
| --- | --- | --- | --- |
| Loganic acid | Y=1579932X+3162 | 0.9996 | 0.03~0.92 |
| Swertiamarin | Y=148897X+4724 | 0.9996 | 0.03~0.91 |
| Gentiopicroside | Y=1090520X+2324 | 0.9997 | 0.03~0.77 |
| Sweroside | Y=1496667X+4332 | 0.9997 | 0.03~0.94 |
| Isoorientin | Y=2142161X+7519 | 0.9998 | 0.03~0.92 |
| Isoscoparin-2″-O-β-D-glucopyranoside | Y=1468841X+4001 | 0.9999 | 0.03~0.92 |

**Table S3** Total yields per plant of six bioactive compounds in flowers of G. lawrencei var. farreri under S15 and S20 treatments

| Compound | S15 Concentration  (mg/g) | S20 Concentration  (mg/g) | S15 Total Yield (mg/plant) | S20 Total Yield (mg/plant) |
| --- | --- | --- | --- | --- |
| Loganic acid | 17.50 ± 0.10 | 59.19 ± 0.03 | 6.17 ± 0.31 | 7.66 ± 0.39 |
| Swertiamarin | 10.98 ± 0.29 | 34.23 ± 0.03 | 3.87 ± 0.20 | 4.43 ± 0.23 |
| Gentiopicroside | 35.19 ± 0.20 | 42.34 ± 0.04 | 12.41 ± 0.63 | 5.48 ± 0.28 |
| Sweroside | 1.87 ± 0.01 | 2.29 ± 0.01 | 0.66 ± 0.03 | 0.30 ± 0.02 |
| Isoorientin | 14.40 ± 0.10 | 8.68 ± 0.12 | 5.08 ± 0.26 | 1.12 ± 0.08 |
| Isoscoparin-2″-O-β-D-glucopyranoside | 7.18 ± 0.24 | 3.32 ± 0.26 | 2.53 ± 0.13 | 0.43 ± 0.06 |

**Note**: Data are presented as mean ± SD (n = 3). Total yield per plant (mg/plant) was calculated as concentration (mg/g) × mean dry weight per plant (g/plant).
